# IFN-γ Producing Th1 Cells Induce Different Transcriptional Profiles in Microglia and Astrocytes

**DOI:** 10.3389/fncel.2018.00352

**Published:** 2018-10-10

**Authors:** Chittappen K. Prajeeth, Oliver Dittrich-Breiholz, Steven R. Talbot, Philippe A. Robert, Jochen Huehn, Martin Stangel

**Affiliations:** ^1^Department of Neurology, Clinical Neuroimmunology and Neurochemistry, Hannover Medical School, Hannover, Germany; ^2^Research Core Unit Genomics, Hannover Medical School, Hannover, Germany; ^3^Institute for Laboratory Animal Science and Central Animal Facility, Hannover Medical School, Hannover, Germany; ^4^Experimental Immunology, Helmholtz Centre for Infection Research, Braunschweig, Germany; ^5^Department of Systems Immunology, Braunschweig Integrated Centre of Systems Biology, Helmholtz Centre for Infection Research, Braunschweig, Germany; ^6^Center for Systems Neuroscience, Hannover, Germany

**Keywords:** microglia, astrocytes, Th1 cells, cytokines, interferon-γ

## Abstract

Autoreactive T cells that infiltrate into the central nervous system (CNS) are believed to have a significant role in mediating the pathology of neuroinflammatory diseases like multiple sclerosis. Their interaction with microglia and astrocytes in the CNS is crucial for the regulation of neuroinflammatory processes. Our previous work demonstrated that effectors secreted by Th1 and Th17 cells have different capacities to influence the phenotype and function of glial cells. We have shown that Th1-derived effectors altered the phenotype and function of both microglia and astrocytes whereas Th17-derived effectors induced direct effects only on astrocytes but not on microglia. Here we investigated if effector molecules associated with IFN-γ producing Th1 cells induced different gene expression profiles in microglia and astrocytes. We performed a microarray analysis of RNA isolated from microglia and astrocytes treated with medium and Th-derived culture supernatants and compared the gene expression data. By using the criteria of 2-fold change and a false discovery rate of 0.01 (corrected *p* < 0.01), we demonstrated that a total of 2,106 and 1,594 genes were differentially regulated in microglia and astrocytes, respectively, in response to Th1-derived factors. We observed that Th1-derived effectors induce distinct transcriptional changes in microglia and astrocytes in addition to commonly regulated transcripts. These distinct transcriptional changes regulate peculiar physiological functions, and this knowledge can help to better understand T cell mediated neuropathologies.

## Introduction

Under physiological conditions the CNS is inaccessible to T cells and other peripheral immune cells. Nevertheless, to minimize the potential risk from invading pathogens, surveillance of distinct anatomical sites of the CNS by T cells is a common phenomenon. Although activation of T cells in response to invading pathogens can help in the resolution of infection, ensuing neuroinflammation can be highly deleterious (Klein and Hunter, 2017). This is also the case in certain neurological autoimmune diseases, where activation of autoreactive T cells can cause irreversible neural tissue damage (Goverman, 2009; Fletcher et al., 2010). Therefore, control of T cell entry and activation is vital to regulate protective and pathological responses within the CNS. While the compact blood brain barrier (BBB) restricts the entry of the T cells into the CNS, their activation is regulated by resident glial cells such as microglia and astrocytes.

Under steady-state conditions, microglia, and astrocytes are key regulators of neural homeostasis owing to their more neurotrophic and less pro-inflammatory properties (Colonna and Butovsky, 2017; Liu et al., 2017). Nevertheless, under pathological conditions induced by trauma, infection or infiltration of autoimmune cells this balance is disturbed, leading to their activation and hence altering their phenotype and function which is either beneficial or destructive. It is believed that in disorders such as multiple sclerosis (MS), autoreactive CD4^+^ T helper cell subsets directed against neural tissue antigens find their way into the CNS through leaky BBB (Fletcher et al., 2010; Sallusto et al., 2012). The knowledge gained from *in vitro* studies and from animal models such as experimental autoimmune encephalomyelitis (EAE) suggests that effector T helper cells provide factors that induce a pro-inflammatory phenotype to microglia and astrocytes, and this step is crucial in driving the neuropathology of MS (McQuillan et al., 2010; Murphy et al., 2010; Prajeeth et al., 2014, 2017, 2018; Lassmann and Bradl, 2017). Among CD4^+^ T helper cells, interferon-γ (IFN-γ)-producing Th1 and interleukin-17 (IL-17)-producing Th17 cells are key players in MS pathogenesis. Our previous work has demonstrated that effector molecules secreted from Th1 and Th17 cells act on distinct targets within the CNS. We have shown that effector molecules released by Th1 cells activate both microglia and astrocytes, whereas Th17-derived effector molecules directly activate only astrocytes and not microglia (Prajeeth et al., 2014, 2017). The reason for this is still unclear. However, it is believed that astrocytes are better equipped with the machinery to respond to Th17-derived effector molecules (Kang et al., 2010).

Microglia and astrocytes are associated with diverse functions within the CNS and they can both drive neuroinflammation with a varying degree of severity. It is known that Th1 effectors can induce a proinflammatory response both in microglia and astrocytes (McQuillan et al., 2010; Prajeeth et al., 2014, 2017). However, it is poorly understood if factors released by Th1 cells have any other distinct influence on the function of microglia and astrocytes. In this study we compared the differentially expressed genes (DEGs) of microglia and astrocytes after stimulation with Th1-derived supernatants to get a better understanding of the functional changes induced by Th1-derived effector molecules.

## Methods

### Ethics statement

C57BL/6 mice were housed and bred under specific-pathogen-free conditions in the central animal facility of Hannover Medical School (MHH), Hannover, Germany. All research and animal care procedures were approved by the Review Board of the care for Animals Subjects of the district government (Lower Saxony, Germany) and performed according to international guidelines on the use of laboratory animals (Nicklas et al., 2002).

### *In vitro* differentiation of Th1 cells

Naïve CD4^+^CD25^−^ cells from C57BL/6 mice were differentiated *in vitro* into Th1 cells as previously described (Prajeeth et al., 2014) with slight modifications. Briefly, after enrichment of CD4^+^ T cells from spleen and lymph nodes using CD4^+^ T cell enrichment kit (BD biosciences) naïve CD4^+^CD62L^hi^CD25^−^ cells were sort purified using MoFlo (Beckman- Coulter) or FACSAria (BD biosciences). Cells (5.0 × 10^5^/ml) were stimulated with plate-bound anti-CD3 (2 μg/ml) and anti-CD28 (2 μg/ml) in 12-well plates (Corning Life Science, Acton, MA) in complete IMDM (IMDM, 10% fetal calf serum (FCS), 1 mM Sodiumpyruvate 50 μM β-mercaptoethanol, 25 mM HEPES and non-essential amino acids) supplemented with either Th1-polarizing factors IL-12 (20 ng/ml), anti-IL-4 (10 μg/ml). After 6 days culture, Th1 cells were harvested and restimulated in 12-well plates coated with anti-CD3 and anti-CD28 antibodies for 6 h. Supernatants devoid of cells were collected and stored at −80°C until further use. Purity of Th1 cultures was assessed by intracellular staining after PMA/iono restimulation, with appropriate dilutions of anti-IFNγ (clone: XMG1.2) and anti-IL17 (clone: TC11-18H10.1) antibodies (Supplementary Figure 1D).

### Cytokine profiling of Th1 culture supernatants

Cytokine profiles of supernatants from anti-CD3/CD28 restimulated Th1 cultures were analyzed using LEGENDplex^TM^ mouse Th cytokine (13-plex) multi-analyte flow assay kit (Biolegend) according to the manufacturer's instructions. Undiluted and 1:30 dilution of the culture supernatants was used for measurements. The concentrations of various cytokines in Th1 culture supernatants are presented in Supplementary Figure 1E.

### Preparation of primary mouse mixed glial cells

Primary cultures of mixed glial cells were prepared from brains of postnatal 1–3 days old C57BL/6 neonatal mice as previously described (Prajeeth et al., 2014). Briefly, the brains were freed from meninges, and digested enzymatically with 0.1% trypsin (Sigma-Aldrich) and 0.25% DNAse (Roche, Mannheim, Germany). Single cell suspensions obtained from the digested brains were seeded into poly-L-lysine-coated T 75 mm^2^ culture flasks in medium consisting of DMEM + L-Glutamine + 4.5 g/L D-Glucose (Gibco®, Darmstadt, Germany) supplemented with 10% FCS, 50 U/ml penicillin, and 50 μg/ml streptomycin (all Biochrom AG, Berlin, Germany). After 24 h all media containing cell debris was removed and fresh media was added. Medium was changed every fourth day and the microglia were harvested at day 9–11 by shaking the flask at 37°C and 180 rpm for 30 min on an orbital shaker. Microglia harvested in this manner was highly pure as tested by high percentage of CD11b^+^ cells on flow cytometry (Supplementary Figure 1A). Remaining microglia and oligodendrocyte precursor cells were eliminated by overnight shaking at 37°C and 170 rpm in an orbital shaker, followed by cytosine arabinoside (AraC; 10 μM; Sigma Aldrich) treatment for 3 days. Astrocytes were harvested from the culture flasks by mild trypsinization and were replated into 6-well plates at a density of 3 × 10^5^ cells per well. Confluent astrocyte cultures were shaken at 37°C and 180 rpm in an orbital shaker for 4 h to eliminate any remaining contaminating microglia. Astrocytes obtained in this way were referred to as highly enriched with <3% microglial contamination (CD11b^+^ cells) as assessed by flow cytometry (Supplementary Figure 1B). Immunofluorescence staining with antibodies against glial fibrillary acidic protein (GFAP) and Ibal confirm that astrocyte cultures generated with this protocol are highly pure with mostly GFAP^+^ cells (astrocytes) and very few contaminating Iba1^+^ cells (microglia; Supplementary Figure 1C).

### Stimulation of microglia and astrocytes with Th1-derived supernatants

After harvesting, 5 × 10^5^ microglia were plated in 6-well plates and incubated for 24 h to regain their resting phenotype. Similarly confluent astrocyte cultures as obtained in the above procedure were treated for 16 h with Th1-derived culture supernatants diluted with an equal volume of fresh complete DMEM. In all experiments, medium controls refer to T cell culture medium (complete IMDM) collected, frozen, and diluted with an equal volume of complete DMEM just before treatment of astrocytes.

### Microarray

The microarray analysis of microglia has been previously published (Prajeeth et al., 2014) and the data set is available under Gene Expression Omnibus (GEO) accession number: GSE45384 (http://www.ncbi.nlm.nih.gov/geo/query/acc.cgi?acc=GSE45384).

RNA was isolated from astrocytes using the RNeasy Micro kit (Qiagen) according to the manufacturer's instructions. Quality and integrity of the total RNA was controlled on an Agilent Technologies 2100 Bioanalyzer (Agilent Technologies; Waldbronn, Germany).

The Microarray utilized in this study represents a refined version of the Whole Mouse Genome Oligo Microarray 4x44K v2 (Design ID 026655, Agilent Technologies), called “048306On1M” (Design ID 066423) developed by the Research Core Unit Genomics (RCUG) of the Hannover Medical School. Microarray design was created at Agilent's eArray portal using a 1 × 1 M design format for mRNA expression as template. All non-control probes of design ID 026655 have been selected to be printed four times within a region comprising a total of 1,81,560 Features (170 columns × 1,068 rows). Four of such regions were placed within one 1 M region giving rise to four microarray fields per slide to be hybridized individually (Customer Specified Feature Layout). Control probes required for proper Feature Extraction software operation were determined and placed automatically by eArray using recommended default settings.

Synthesis of Cy3-labeled cRNA was performed with the “Quick Amp Labeling kit, one color” (#5190-0442, Agilent Technologies) using 500 ng of total RNA as input and according to the manufacturer's recommendations, except that reaction volumes were halved. cRNA fragmentation, hybridization, and washing steps were carried-out exactly as recommended in the “One-Color Microarray-Based Gene Expression Analysis Protocol V5.7.” cRNA fragmentation, hybridization and washing steps were carried-out as recommended in the “One-Color Microarray-Based Gene Expression Analysis Protocol V5.7.” Slides were scanned on the Agilent Micro Array Scanner G2565CA (pixel resolution 3 μm, bit depth 20). Data extraction was performed with the “Feature Extraction Software V10.7.3.1” using the extraction protocol file “GE1_107_Sep09.xml,” except that “Multiplicative detrending” algorithm was inactivated.

In order to comparatively analyze our previously generated, published microglia data (GEO accession number GSE45384) with analogously generated data from astrocytes in the current study, raw data from both studies were reprocessed and normalized together, as follows:

Starting with the main output of the Feature Extraction Software (txt-files), measurements of contained on-chip replicates were averaged using the geometric mean of processed intensity values of the green channel, “gProcessedSignal” (gPS) to retrieve one resulting value per unique non-control probe. Single Features were excluded from averaging, if they (i) were manually flagged, (ii) were identified as Outliers by the Feature Extraction Software, (iii) lay outside the interval of “1.42 × interquartile range” regarding the normalized gPS distribution of the respective on-chip replicate population, or, (iv) showed a coefficient of variation of pixel intensities per Feature that exceeded 0.5.

Averaged gPS values were normalized by quantile normalization followed by global linear scaling. For the latter approach, all quantile normalized gPS values of one sample were multiplied by an array-specific scaling factor. This factor was calculated by dividing a “reference 75th Percentile value” (set as 1500 for the whole series) by the 75th Percentile value of the particular Microarray to be normalized (“Array i” in the formula shown below). Accordingly, finally normalized gPS values for all samples (microarray data sets) were calculated by the following formula:

Finally normalized gPSArray i = quantile normalized gPSArray i × (1,500/75th PercentileArray i).

Finally, a lower intensity threshold (surrogate value) was defined based on intensity distribution of negative control features. This value was fixed at 15 finally normalized gPS units. All of those measurements that fell below this intensity cutoff were substituted by the respective surrogate value of 15. For subsequent analysis and visualization accordingly processed data were imported into GeneSpring GX software, version 13.1.1 (Agilent Technologies Inc., Santa Clara, CA). Normalized values were imported as single-color data and were log2-transformed according to the default import procedure. No additional data transformation or normalization was applied during data import.

A moderated *T*-test was applied to identify differentially expressed mRNAs, using a corrected *p*-value cut off at 0.01 (Benjamini Hochberg correction; false discovery rate of 0.01) and showing an additional fold change of more than 2-fold. Data were analyzed through the use of Ingenuity Pathway Analysis (IPA) (Qiagen) to identify and compare pathways that were altered by Th1-derived effectors in astrocytes and microglia. Z-scores were used to determine if pathways have significantly more activated predictions than they have inhibited predictions. Z-score values were considered significant if >2 or <−2.

The new astrocyte microarray and reprocessed microglia data (GSE45384) set is available under GEO accession number: GSE113579.

https://www.ncbi.nlm.nih.gov/geo/query/acc.cgi?acc=GSE113579.

### General computation and plotting

The computation and visualization was done using the R (v.3.4.1 2017-06-30) software with the following packages on a four kernel 64-bit machine.

[1] R Core Team (2017). R: A language and environment for statistical computing. R Foundation for Statistical Computing, Vienna, Austria. https://www.R-project.org/.

[2] Hadley Wickham and Jennifer Bryan (2017). readxl: Read Excel Files. R package version 1.0.0., https://CRAN.R-project.org/package=readxl.

[3] Raivo Kolde (2015). pheatmap: Pretty Heatmaps. R package version 1.0.8., https://CRAN.R-project.org/package=pheatmap.

[4] Erich Neuwirth (2014). RColorBrewer: ColorBrewer Palettes. R package version 1.1-2., https://CRAN.R-project.org/package=RColorBrewer.

### Heat maps

An Excelfile was created with a list of 90 genes which were functionally characterized as cytokines and growth factors in ingenuity knowledge base and filtered from the total list of 3,170 DEGs in astrocytes and microglia. The FC values were log_2_-transformed for better scaling of extreme values. The Excelfile was then imported into R. With the colorRamp function a color gradient between blue and red was generated and centered at log_2_ (FC) = 0 = “white.” The mean of four columns of astrocyte/microglia data was then calculated and displayed using the pheatmap function. A fold-change cut-off of 6 was introduced for better color scaling of the data. All log_2_(FC) values above 6 will be considered as 6 in the resulting heat map.

### Volcano plots

Volcano plots were constructed by plotting –log_10_ (*p*-value) against log_2_ (fold change) values of all the 30028 transcripts representing all the genes in the array. Thresholds were set to *p* < 0.01 (red), log_2_(FC) >2 (orange), and *p* < 0.01 and log_2_(FC) >2 (green).

### Scatter plot (four-way plot)

All DEGs (3,170 genes) filtered from astrocytes and microglia using above criteria (FC ≥2.0 and *p* ≤ 0.01) were used for generating a four-way plot. For certain genes that did not satisfy the significance *p* < 0.05 in a cell type the values were adjusted to FC = 1. The FC values were log_2_-transformed so that downregulated genes still show negative signs. Then values were filtered for different log_2_(FC) thresholds. The filtered number of genes was then annotated into the corresponding area/corner of the plot.

### Real-time reverse transcriptase polymerase chain reaction (RT-PCR)

RNA was isolated from the using the RNeasy Mini kit (Qiagen) according to the manufacturer's instructions. Equal amounts of RNA (560–1,700 ng) were subsequently transcribed into cDNA with the High Capacity cDNA Reverse Transcription Kit (#4368814; Applied Biosystems®; Life Technologies GmbH, Darmstadt, Germany). For gene expression analysis quantitative real-time PCR was performed using the StepOne™ Real-Time PCR System and appropriate TaqMan probes (Applied Biosystems, Supplementary Table 2). The ΔΔCt method was applied to determine differences in the expression between astrocytes treated with medium and Th1 supernatants. Changes in mRNA expression levels were calculated after normalization to hypoxanthin phosphoribosyltransferase (Hprt1) and glyceraldehyde 3-phosphate dehydrogenase (Gapdh).

### Immunofluoresence and microscopy

For immunofluorescence, astrocytes plated on coverslips were stained with primary mouse monoclonal anti-glial fibrillary acidic protein (GFAP; GA5; Millipore) and polyclonal rabbit Iba1 (Wako) antibodies in 0.3% Triton in PBS for 2 h at room temperature (RT). After thorough washing, they were incubated with Alexa Fluor 488 conjugated goat-anti mouse and Alexa Fluor 555 conjugated goat-anti rabbit secondary antibodies (both from Molecular probes®, Life technologies^TM^) in 0.3% Triton PBS for 1 h at RT. After washing slides were mounted with ProLong^TM^ Gold antifade reagent with DAPI (Molecular probes®, Life technologies™). Images were taken using microscope (Olympus BX41) with camera.

## Results

### Transcriptional changes in microglia and astrocytes in response to Th1-derived effectors

**Figure 1:**
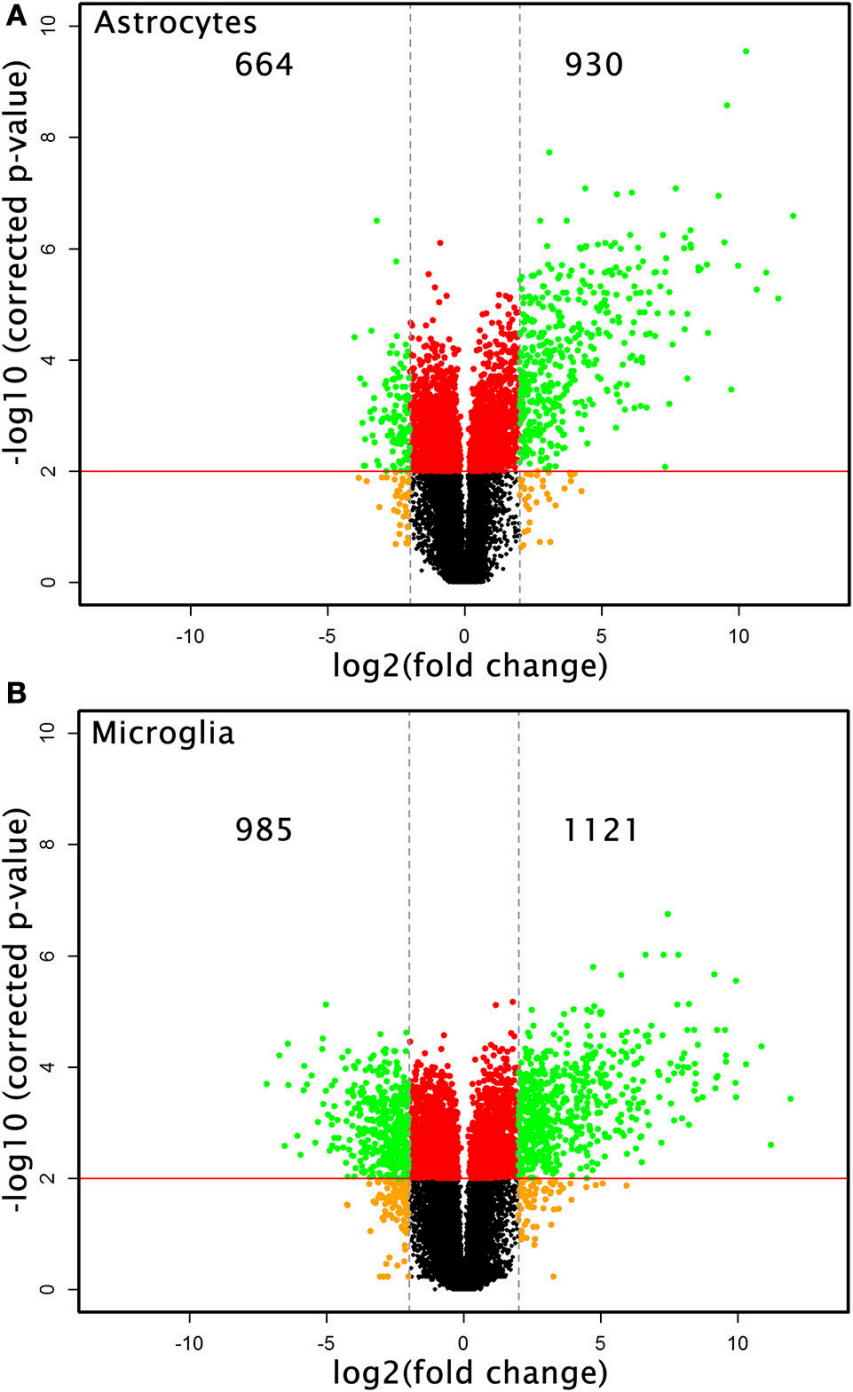
Transcriptional changes induced by Th1-derived effector molecules in microglia and astrocyte. Volcano plot shows the overall picture of differentially expressed genes (DEG) in astrocytes **(A)** and microglia **(B)** in response to Th1-derived effectors. Microarray analysis was performed on the RNA isolated from these cell types treated with medium or Th1-derived culture supernatants. Data generated from four independent experiments (*n* = 4). Fold changes were determined by normalizing the expression induced by Th1 treatment to their respective medium controls. Fold changes ≥2.0 and false discovery date of 0.01(corrected *p* < 0.01) was used as criteria to enrich DEGs.

Microarray analysis of total RNA isolated from microglia and astrocytes treated with Th1-derived culture supernatants show significant changes in the transcriptome of both microglia and astrocytes. We used fold change (FC) ≥2.0 and a false discovery rate (FDR) of 0.01 (corrected *p* < 0.01) to identify 2,106 and 1,594 DEGs in microglia and astrocytes, respectively, in response to Th1-derived effectors. As shown in Figure 1, Th1-derived effectors enhanced the expression of significant proportions of genes in both microglia (1,121 genes) and astrocytes (930 genes) compared to respective medium-treated controls. Similarly, expression of several genes was down-regulated in both the cells types (985 genes in microglia and 664 genes in astrocytes; Figure 1). We listed the top 10 genes that were up- and down-regulated in each cell type (Table 1). Highly upregulated genes in one cell type predominantly followed similar direction of regulation also in the other cell type although with variable induction levels. *Cxcl9, Gbp6, Serpina3g*, and *Tgtp1/Tgtp2* were among the top upregulated genes in both cell types. Interestingly, a certain extent of cell specificity was observed among the top downregulated genes. Most of the genes downregulated in astrocytes were not regulated in microglia and those that were highly downregulated in microglia were either moderately or not regulated in astrocytes.

**Table 1:** Genes highly regulated in microglia and astrocytes in response to Th1-derived effectors.

**Entrez Gene ID**	**Gene symbol**	**log_2_ FC**	**log_2_ FC (Microglia)**
**(A) ASTROCYTES**			
**TOP UPREGULATED GENES**			
17329	Cxcl9	11.98624	11.21897
24108	UBD	11.44265	5.77813
17472	GBP6	10.99735	10.86459
225594	Gm4841	10.26361	9.144518
20306	Ccl7	9.730344	5.962017
74481	BATF2	9.566627	6.860566
100039796	Tgtp1/Tgtp2	9.476637	9.217602
215900	CALHM6	9.266997	6.631192
16149	CD74	8.886718	5.191365
238393	Serpina3g	8.834784	11.93777
**TOP DOWNREGULATED GENES**			
20606	SSTR2	−4.026092	nr
93732	ACOX2	−3.808076	nr
107585	DIO3	−3.733354	nr
15446	HPGD	−3.663572	−6.536193
100559	UGT2B28	−3.657526	nr
17863	MYB	−3.640042	nr
12818	COL14A1	−3.630289	nr
53318	Pdlim3	−3.406401	nr
54612	SFRP5	−3.295135	nr
381107	TMEM232	−3.286881	nr
**Entrez Gene ID**	**Gene symbol**	**log**_2_ **FC**	**log**_2_ **FC (Astrocytes)**
**(B) MICROGLIA**			
**TOP UPREGULATED GENES**			
20715	Serpina3g	11.93777	8.834784
17329	Cxcl9	11.21897	11.98624
17472	GBP6	10.86459	10.99735
66857	PLBD1	10.30093	4.26122
20307	Ccl8	9.944667	7.576167
110454	Ly6a	9.927621	7.475109
16161	IL12RB1	9.578259	6.742087
108116	SLCO3A1	9.568786	2.002523
384059	Tlr12	9.271939	6.427522
100039796	Tgtp1/Tgtp2	9.217602	9.476637
**TOP DOWNREGULATED GENES**			
101488	SLCO2B1	−7.188292	−3.105208
321019	GPR183	−6.715591	−2.320636
15446	HPGD	−6.536193	−3.663572
69574	CMBL	−6.407914	−1.909581
56811	DKK2	−6.055695	nr
13051	CX3CR1	−5.952334	nr
56792	STAP1	−5.834989	nr
117590	ASB10	−5.819413	nr
71738	MAMDC2	−5.717594	−1.296311
78771	MCTP1	−5.53596	nr

### Identification of upstream regulators, canonical pathways, and functions from DEGs

**Figure 2:**
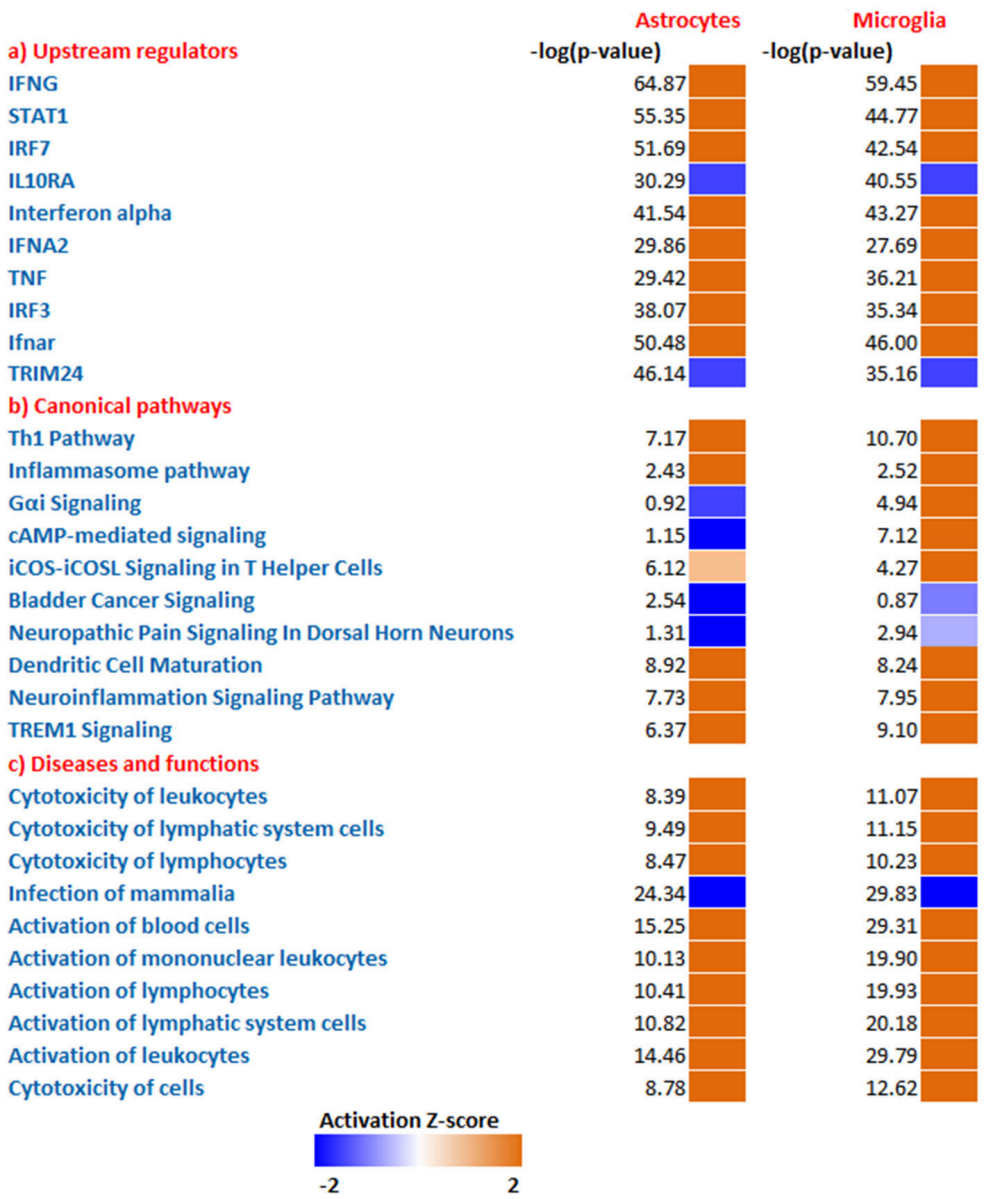
Comparative analysis all DEGs enriched from microglia and astrocytes. Using IPA software from Ingenuity systems, we performed this comparative analysis to determine top **(a)** upstream regulators **(b)** canonical pathways **(c)** Disease and functions from the enriched gene sets. The heat maps were sorted using the trend and activation Z-score. Orange and blue boxes represent positive and negative Z-scores, respectively.

To predict the molecules acting upstream and responsible for inducing the transcriptional changes in microglia and astrocytes, we performed an upstream regulator analysis using the IPA software. Based on the activation Z-score we obtained almost similar top hits as upstream regulators in both microglia and astrocytes (Figure 2a; Table 2). IFN-γ and STAT1 were among the top upstream regulators in microglia and astrocytes (Table 2). This was not surprising as IFN-γ was detected in very high concentrations in our Th1-derived culture supernatants and is long known as an effector cytokine mediating effects of Th1 cells (Prajeeth et al., 2014). In accordance with these findings, we have also observed upregulation of several IFN-γ responsive genes in the transcriptome in both microglia and astrocytes. These include genes encoding guanylate *Gbp2, Irf1, CD274, Ifi47*, and others (Supplementary Table 1). We also found signatures of an IFN-α receptor (IFNAR)-mediated response as we detect *Irf3* and *Irf7* among the top upstream regulators. It must be noted that these transcriptional regulators are induced by type I IFN and also further control the expression of interferon-stimulated genes. Activated microglia and astrocytes are known to produce type I IFN.

**Table 2:** IPA upstream analysis; top10 upstream regulators listed for astrocytes and microglia.

**Upstream regulator**	**Molecule type**	**Predicted activation state**	**Activation z-score**	***p*-value of overlap**
**(A) ASTROCYTES**				
IFNG	Cytokine	Activated	12.513	1.36E-65
STAT1	Transcription regulator	Activated	8.875	4.43E-56
IRF7	Transcription regulator	Activated	8.32	2.04E-52
Ifnar	Group	Activated	7.487	3.32E-51
TRIM24	Transcription regulator	Inhibited	−7.17	7.32E-47
poly rI:rC-RNA	Biologic drug	Activated	9.514	1.06E-45
Interferon alpha	Group	Activated	8.228	2.88E-42
lipopolysaccharide	Chemical drug	Activated	9.334	3.07E-41
IRF3	Transcription regulator	Activated	7.422	8.60E-39
**(B) MICROGLIA**				
Lipopolysaccharide	Chemical drug	Activated	9.95	6.14E-61
IFNG	Cytokine	Activated	11.559	3.56E-60
Ifnar	Group	Activated	7.234	1.01E-46
STAT1	Transcription regulator	Activated	7.957	1.70E-45
poly rI:rC-RNA	Biologic drug	Activated	8.902	1.34E-44
Interferon alpha	Group	Activated	7.558	5.36E-44
IRF7	Transcription regulator	Activated	8.381	2.86E-43
IL10RA	Transmembrane receptor	Inhibited	−9.492	2.83E-41
TNF	Cytokine	Activated	6.79	6.11E-37
IRF3	Transcription regulator	Activated	7.534	4.54E-36

We further determined canonical signaling pathways enriched based on the DEGs filtered from microglia and astrocytes. Among the pathways that attained highest activation Z-scores microglia and astrocytes share some common pathways that are likely to influence inflammation and T cell activation. Nevertheless, we have also detected certain pathways that attained contrasting Z-scores in microglia and astrocytes. For instance, Gαi/cAMP signaling attained negative Z-score in astrocytes for the reason that several genes known to be associated with this pathway were downregulated in astrocytes. On the other hand Gαi/cAMP signaling had a positive activation Z-score in microglia as majority of genes known to be associated with this pathway showed upregulation (Figure 2b; Table 3).

**Table 3:** Top 10 canonical pathways identified based on all DEGs in astrocytes and microglia.

**Ingenuity canonical pathways**	**–log(*p*-value)**	**Ratio**	**z-score**
**(A) ASTROCYTES**			
Antigen presentation pathway	1.27E+01	4.74E−01	NaN
Type I diabetes mellitus signaling	8.99E+00	2.25E−01	2.138
Dendritic cell maturation	8.92E+00	1.76E−01	4.352
OX40 signaling pathway	8.61E+00	2.42E−01	−1.342
Altered T cell and B cell signaling in rheumatoid arthritis	7.95E+00	2.33E−01	NaN
Neuroinflammation signaling pathway	7.73E+00	1.38E−01	4.111
Death receptor signaling	7.68E+00	2.26E−01	1.964
Activation of IRF by cytosolic pattern recognition receptors	7.56E+00	2.70E−01	1.213
Th1 pathway	7.17E+00	1.85E−01	2.294
Allograft rejection signaling	7.02E+00	2.26E−01	NaN
**(B) MICROGLIA**			
Th1 and Th2 activation pathway	1.11E+01	2.38E−01	NaN
Th1 pathway	1.07E+01	2.67E−01	3.053
Altered T cell and B cell signaling in rheumatoid arthritis	9.44E+00	3.00E−01	NaN
G-protein coupled receptor signaling	9.41E+00	1.92E−01	NaN
T helper cell differentiation	9.38E+00	3.29E−01	NaN
Role of pattern recognition receptors in recognition of bacteria and viruses	9.23E+00	2.48E−01	2.6
Antigen presentation pathway	9.22E+00	4.47E−01	NaN
TREM1 signaling	9.10E+00	3.20E−01	3.266
Colorectal cancer metastasis signaling	8.71E+00	1.94E−01	1.64
Dendritic cell maturation	8.24E+00	2.07E−01	3.569

Analysis of transcriptional changes in microglia and astrocytes in the context of diseases and functions reveal that activation of leukocytes and cytotoxicity of target cells are among the common functions influenced by Th1-activated microglia and astrocytes. Since many of the genes were induced in response to interferons the enriched gene set also gives clues of likely antiviral responses of microglia and astrocyte (Figure 2c).

### Distinct transcriptional profile induced by Th1-derived effectors in microglia and astrocytes

**Figure 3:**
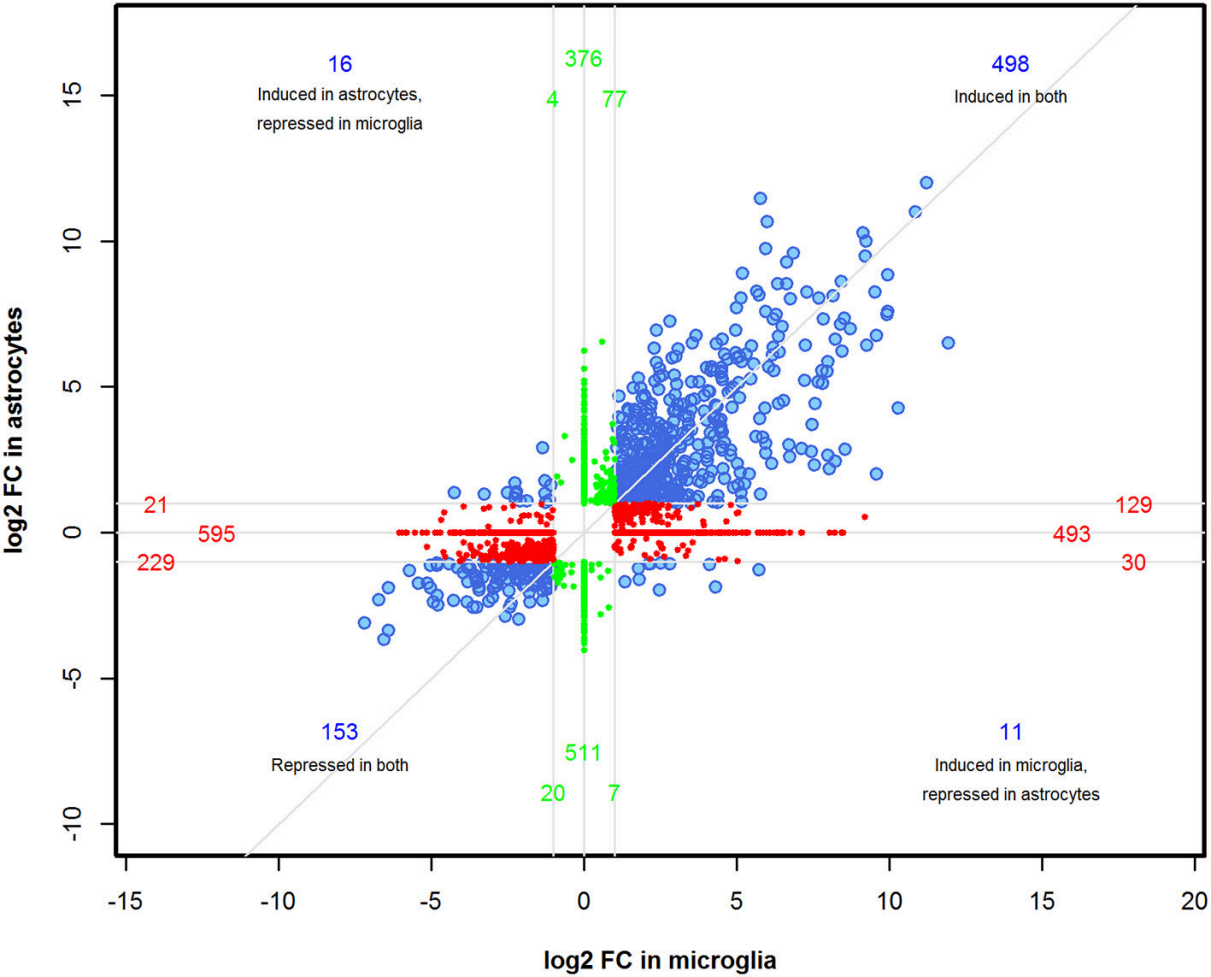
Identification of distinct transcriptional changes induced by Th1-derived effectors in microglia and astrocytes. A four-way plot was generated using 3170 DEGs enriched between microglia and astrocytes based on fold changes ≥2.0 and a false discovery rate of 0.01 (corrected *p* < 0.01) selection criteria. FC value was adjusted to 1 for those genes where corrected *p* > 0.05 in either of the cell type. log_2_ (fold change) values of astrocytes was plotted against microglia. Blue dots represent common regulated genes, red dots are genes more specifically regulated in microglia and the green dots indicate astrocyte specific genes.

We performed a comparison analysis of DEGs induced in microglia and astrocytes by Th1-derived effectors in order to identify if any specific gene expression pattern emerged in these cell types. A total of 3,170 genes were identified between astrocytes and microglia with FDR < 0.01 and ≥2-fold change. However, not all genes in either cell type fulfilled the selection criteria. Therefore, the fold change value was adjusted to FC = 1 for those genes where corrected *p*-value was >0.05. As demonstrated in the four-way plot (Figure 3), we identified 498 genes (~15%) induced and 153 genes (~5%) repressed were common for both microglia and astrocytes. Interestingly, we also found expression of several genes that were specifically regulated either in astrocytes or microglia. For instance, in astrocytes 396 genes (~12%) that were upregulated are either unchanged or showed downregulation in microglia and 534 genes (~17%) showing upregulation in microglia were either unchanged or show downregulation in astrocytes. Similarly, 632 (~20%) and 529 (~17%) genes were specifically downregulated in microglia and astrocytes, respectively, in response to Th1-derived effectors (Figure 3). This provides clear evidence that Th1-derived effectors induced distinct transcriptional changes in microglia and astrocytes.

### Determining functions governed by DEGs specifically enriched in astrocytes and microglia

As mentioned above, 1,166 and 925 genes were found to be differentially expressed specifically in microglia and astrocytes, respectively, in response to Th1-derived effector molecules. To understand the associated physiological functions we performed IPA analysis on these enriched gene sets. From the top hits obtained based on the high –log (*p*-value), we selected those that were relevant in the context of the CNS functioning. The top canonical pathway specifically induced in microglia was the cAMP-dependent-, G protein-coupled receptor (GPCR)-triggered signaling cascade that plays a crucial role in cell communication. Chemokine receptors are among the popular GPCR and are likely to play a role in activation and migration of cells. The phagosome formation pathway was another top listed canonical pathway that was regulated in microglia (Table 4). Furthermore, the gene encoding MerTK, a functional regulator of myelin phagocytosis (Healy et al., 2016), is specifically upregulated in microglia suggesting that Th1-derived effector molecules are likely to regulate the phagocytic function of microglia. Interestingly, Th1-derived effectors also had some negative effects on certain pathways. Several genes known to regulate the cholesterol biosynthesis pathway were found to be downregulated in microglia and hence were listed among the top negatively regulated pathways in microglia.

**Table 4:** Top canonical pathways regulated by genes specifically up- or downregulated in microglia and astrocytes in response to Th1-derived effectors.

**Ingenuity canonical pathways**	**–log(*p*-value)**	**Molecules**
**(A) ASTROCYTES**		
Axonal guidance signaling	6.16	NTF3,ADAMTS8,RND1,BMP4,SLIT1,MYL2,ITSN1,BDNF,BMP2,CXCL12,SEMA4C,EPHB6,FGFR3, NTNG1,SEMA6D,EPHB1,TUBA8,FGFR4,PLXNB3,GNG12,UNC5C,PAK4,TUBB3,PAK6,ARHGEF15, VEGFC,DPYSL5,ROBO3,PLXND1,PDGFB,WNT10A,EPHB3,EPHA5,BMP7,BMP6,GLI1
Hepatic fibrosis/hepatic stellate cell activation	4.43	COL8A2,CTGF,MYL2,COL2A1,VEGFC,MYH7,IL6,MYH7B,PDGFB,COL26A1,FGF1,COL16A1, COL5A3,HGF,COL4A4,COL11A1,COL9A2,TNFRSF11B
FGF Signaling	3.79	FGFR3,FGF18,FGF9,HGF,FGFR4,FGF14,FGF12,FGF23,CREB5,MAPK11,FGF1
Cellular effects of sildenafil (Viagra)	3.49	SLC4A5,Ppp1r12b,CACNA1S,MYL2,GPR37,CACNG4,ADCY1,GUCY1A2,CACNA1C, MYH7,KCNH2,MYH7B,Gucy1b2
Cardiomyocyte differentiation via bmp receptors	3.45	BMP4,MYL2,BMP2,BMP7,MYH7
Bladder cancer signaling	3.28	FGFR3,FGF18,FGF9,MMP15,FGF14,FGF12,FGF23,VEGFC,MMP17,FGF1
Ephrin receptor signaling	2.78	PAK4,ITSN1,PAK6,ARHGEF15,CXCL12,VEGFC,CREB5,PDGFB,FGF1, EPHB6,EPHB1,EPHB3,EPHA5,GNG12
Corticotropin releasing hormone signaling	2.74	NOS1,CACNA1S,CACNG4,BDNF,ADCY1,MEF2A,GUCY1A2,CACNA1C,CREB5, MAPK11,GLI1,Gucy1b2
Actin cytoskeleton signaling	2.53	PAK4,MYL2,PAK6,FGF9,FGF14,MYH7,MYH7B,PDGFB,FGF1,FGFR3, Ppp1r12b,FGF18,FGFR4,FGF12,FGF23,GNG12
BMP signaling pathway	2.51	SOSTDC1,BMP4,SMAD9, BMP2,BMP7,BMP6,MAPK11,SMURF1
**(B) MICROGLIA**		
G-Protein coupled receptor signaling	8.55	NAPEPLD,HTR2B,RGS18,ADCY4,ADORA3,HTR1D,CHRM3,MPPE1,BRAF,LTB4R,PDE7B,PDE3B, PIK3CG,RGS10,HTR7,RGS14,ADORA2B,RASA1,SRC,FPR2,PDE1C,ADCY9,P2RY13,PLCB4, DUSP9,PRKAR2B,LPAR1,PRKAG2,PIK3R6,S1PR1,P2RY12, PIK3CD,DUSP4,PTGER2,ADCY7,PRKCB
Colorectal cancer metastasis signaling	6.54	MMP7,TGFBR1,MMP14,ADCY4,FZD1,PDGFC,BRAF,GNG11,PIK3CG,TLR1,CTNNB1,WNT5B, RALGDS,Tlr11,SRC,CASP3,GRK3,TYK2,IFNGR1,ADCY9,PRKAR2B,TLR6,MSH6,PRKAG2, PIK3R6,Tlr13,PIK3CD,PTGER2,JAK3,ADCY7
cAMP-mediated signaling	6.28	NAPEPLD,RGS18,ADCY4,ADORA3,HTR1D,CHRM3,MPPE1,BRAF,LTB4R,PDE7B,PDE3B, RGS10,HTR7,ADORA2B,RGS14,SRC,FPR2,PDE1C,ADCY9,P2RY13,DUSP9,PRKAR2B, LPAR1,S1PR1,P2RY12,DUSP4,PTGER2,ADCY7
Phagosome formation	6.14	Tlr11,C5AR1,PRKCQ,FCGR2A,PLCL2,FCGR2B,FCGR1A,PLCD1,PLCB4,Fcrls, SCARA3,PIK3CG,TLR6,TLR1,PIK3R6,Tlr13,PRKCH,PIK3CD,C3AR1,PRKCB
Superpathway of cholesterol biosynthesis	5.83	FDPS,FDFT1,NSDHL,ACAT2,IDI1,MSMO1,TM7SF2,SC5D,CYP51A1
Gαi signaling	5.48	SRC,ADCY4,FPR2,ADORA3,HTR1D,ADCY9,P2RY13,LTB4R,GNG11,PRKAR2B, LPAR1,RGS10,S1PR1,PRKAG2,P2RY12,RGS14,ADCY7,RALGDS
Molecular mechanisms of cancer	4.8	CDKN2A,RAP2A,TGFBR1,ADCY4,CDKN2C,HIF1A,FZD1,BRAF,CCND3,PIK3CG, CTNNB1,RASA1,RALGDS,WNT5B,CDK18,SRC,ARHGEF4,PRKCQ, CASP3,GNA12,TYK2,CDK6,ADCY9,ARHGEF5,PLCB4,MAX,PRKAR2B, CCND2,PRKAG2,PIK3R6,PIK3CD, PRKCH,CDK19,JAK3,ADCY7,PRKCB
Zymosterol biosynthesis	4.34	NSDHL,MSMO1,TM7SF2,CYP51A1
Gustation pathway	3.98	GNAT3,NAPEPLD,ADCY4,CACNB4,P2RX5,CACNA1A,PDE1C,MPPE1, ADCY9,P2RY13,LPAR6,GNG11,PRKAR2B,PDE7B,PDE3B,PRKAG2,P2RY12,ADCY7
eNOS signaling	3.84	PRKCQ,CASP3,ADCY4,CHRM3,PDGFC,HSPA1L,NOSTRIN,ADCY9, LPAR6,PRKAR2B,LPAR1,PIK3CG,PIK3R6,PRKAG2,PRKCH,PIK3CD,ESR1,ADCY7,PRKCB

In contrast, the top canonical pathway regulated in astrocytes is that of erythropoietin-producing human hepatocellular (Eph)/ephrin signaling which is a unique bidirectional signaling pathway involving astrocytes and neurons in the CNS. This signaling is believed to be important in axonal guidance in developing CNS and is likely to play a role in CNS regeneration in adults. Also interesting is that several genes associated with the fibroblast growth factor (FGF) signaling pathway that plays a critical role in neurogenesis appears to be downregulated in astrocytes. Taken together, these results indicate that Th1-derived effectors regulate distinct physiological functions in microglia and astrocytes apart from inducing a common neuroinflammatory phenotype (Table 4).

### Cytokine and growth factor regulation

**Figure 4:**
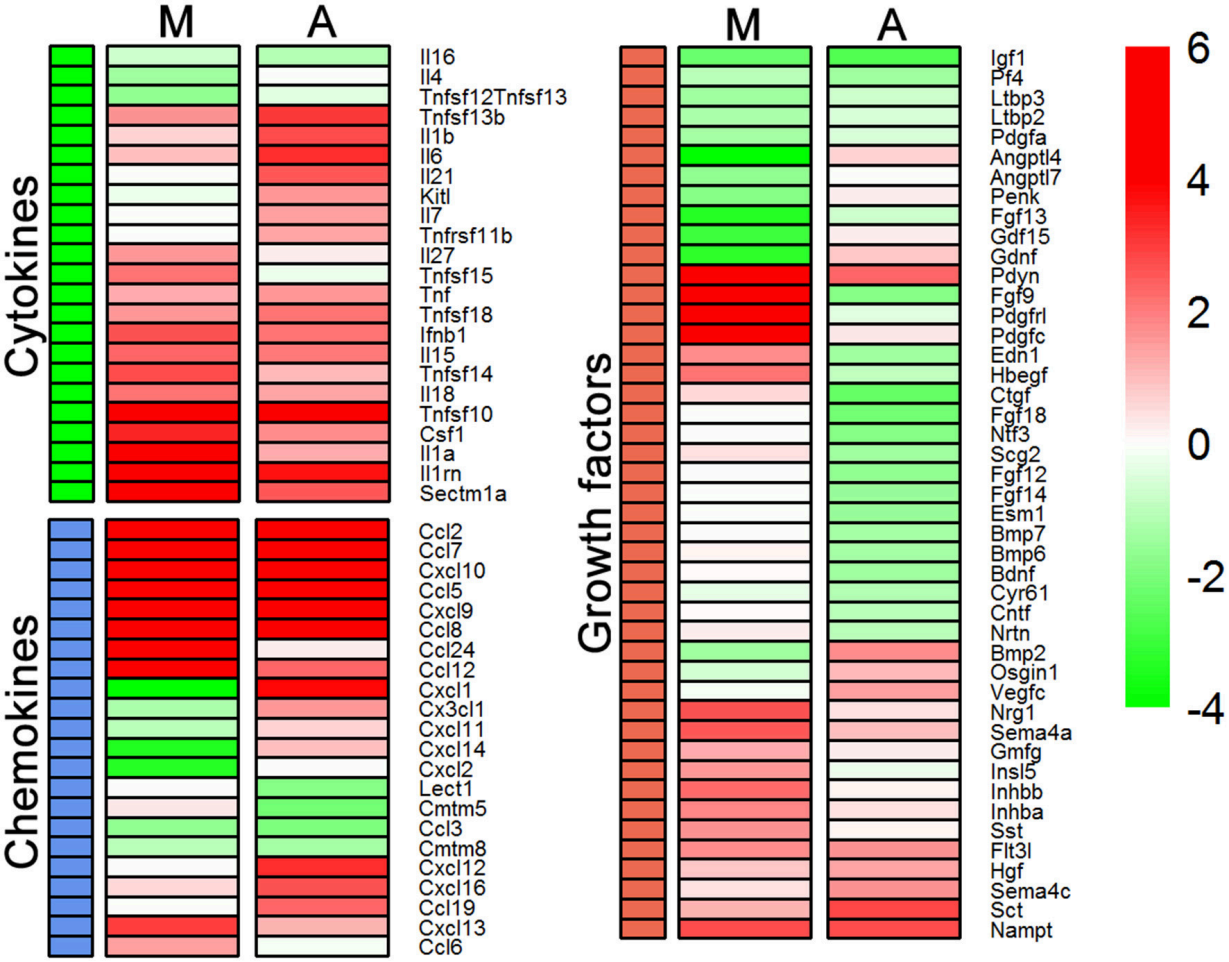
Expression of genes encoding cytokines and growth factors between microglia (M) and astrocytes (A). Heat maps were generated using log_2_ (fold change) values for genes listed for cytokines, chemokines, and growth factors to compare their expression in each cell type.

So far it appears that Th1-derived effectors regulate transcription of those genes in microglia and astrocytes which encode products that govern cell migration, cell morphology, and tissue repair processes. Analysis of regulator effects in IPA has shed light on subtle differences in the task undertaken by these cell types. Most of these mentioned processes are regulated by cytokines and growth factors. Therefore, we selected cytokines and growth factors induced by Th1-derived effectors and compared their expression in microglia and astrocytes. We generated a heat map using the fold change values and observed that astrocytes and microglia demonstrated key differences in the pattern of expression of genes encoding chemokines, cytokines, and growth factors (Figure 4). Genes encoding chemokines such as *Cxcl9, Cxcl10, Ccl5*, and *Ccl2* are upregulated in both microglia and astrocytes. However, *Ccl19, Cxcl12 Cxck16*, and fractalkine (*Cx3cl1*) are specifically upregulated in astrocytes, whereas *Ccl6* and *Ccl24* were the chemokines upregulated in microglia. *Cxcl1* is the only chemokine that was upregulated in astrocytes and downregulated in microglia. Therefore, it is likely that microglia and astrocytes might control movement of cells into and within the CNS by up- or downregulating distinct sets of chemokines. Significant differences in the expression profile of genes encoding cytokines that are capable of altering cell morphology, behavior, and proliferation were also observed between microglia and astrocytes. For instance, gene encoding *Il6*, a key cytokine that drives neuroinflammation, was induced by Th1-derived effectors in astrocytes but not in microglia. On the other hand, *Sectm1a*, a novel co-stimulatory molecule reported to bind to CD7 on T cells and to induce their proliferation (Howie et al., 2013), is upregulated several fold higher in microglia than in astrocytes. Interestingly, expression of genes encoding key growth factors, such as those belonging to the FGF family, *Bdnf* and *Ntf3*, which have a role in neurogenesis and tissue repair, were downregulated in response to Th1-derived effectors in astrocytes but not in microglia. Taken together, Th1-derived effectors induce distinct changes in the transcriptome of microglia and astrocytes that affect vital processes such as chemotaxis, cell proliferation regeneration and repair.

### Kinetics of microglia and astrocyte response to Th1 effectors

**Figure 5:**
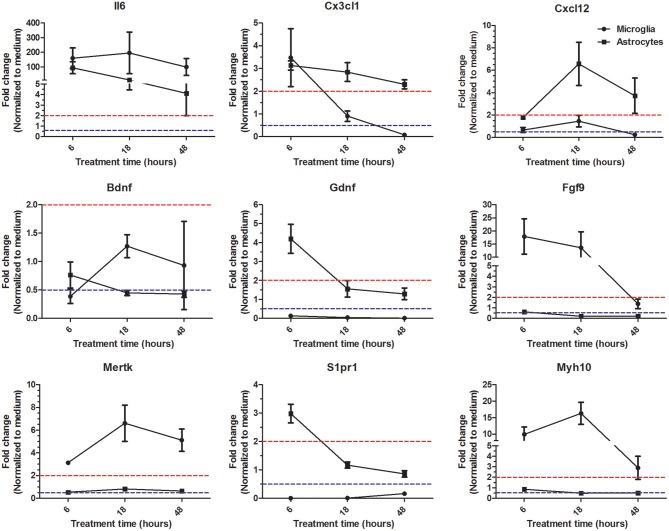
Expression kinetics of genes identified as specifically regulated in microglia and astrocytes. Microglia and astrocytes were treated with Th1 culture supernatants for 6, 18, and 48 h. Expression of given genes was analyzed by RT-PCR. Results are represented as fold changes normalized to respective medium controls. Data above dashed red line indicate upregulation >2-fold and the data below dashed blue line indicate downregulation >2-fold. The data form four independent experiments (*n* = 4) is represented as mean ± SD.

So far our microarray analysis has provided evidence that at a given time point microglia and astrocytes in response to Th1-derived effector molecules exhibited a certain specific gene expression profile that might have influence on distinct cellular processes. However, there is still a possibility that some genes in response to particular stimuli might follow different expression kinetics in different cell types. To address this, we followed expression kinetics of few randomly selected genes that were shown to be specifically regulated in microglia and astrocytes in our microarray analysis. We treated microglia and astocytes for 6, 18, and 48 h with Th1 culture supernatants and assessed the expression of genes shown in Figure 5 using RT-PCR. For majority of the genes tested, we could confirm a similar trend of regulation for 18 h treatment and was consistent with the microarray analysis. Only exception was *Il6* expression which according to microarray analysis has been shown to be specifically upregulated in astrocytes but the RT-PCR analysis reveal that it is highly upregulated in both microglia and astrocytes. Reason for this discrepancy is unclear. Interestingly, few genes displayed certain kinetics in their regulation in response to Th1 treatment. For instance, *Cx3cl1* (fractalkine) is upregulated at early time point (6 h) in microglia and thereafter its expression was downregulated. On the other hand expression of Cx3cl1 was induced early in astrocyte and its expression remained upregulated even at 48 h (Figure 5). Similarly *Gdnf* (glial cell-derived neurotrophic factor) transcript which was downregulated at all time points in microglia was shown to be upregulated by Th1-derived effectors in astrocytes at 6 h time point. These results though confirm the specificity in regulation of certain genes in microglia and astrocytes, it also hints that there might be cell specific gene expression kinetics in response to Th1-derived factors.

## Discussion

Altered behavior of microglia and astrocytes is a common feature in neuroinflammatory diseases such as MS. This is a consequence of their direct interaction with CNS infiltrating immune cells or by sensing the factor(s) released by them. These functions are regulated by proteins encoded by a set of genes and hence understanding the transcriptional changes in response to a particular stimulus can shed light on biological pathways and cell functions influenced by that particular stimulus. Using the approach of unbiased global gene expression profiling we have shown that effector molecules associated with Th1 cells induce robust and distinct transcriptional changes in microglia and astrocytes. While Th1-derived effectors induce a common IFN-γ dependent response in these cell types as demonstrated by our upstream regulator analysis, most valuable information on how they regulate neuroinflammation and subsequent neurodegeneration through glial cells was obtained from the analysis of DEGs that are specifically regulated in microglia and astrocytes.

The induction of a T cell mediated neuropathology such as seen in MS is a highly complex process involving several stages. Presumably the foremost event involves poorly understood primary neuroinflammation that leads to priming of T cells against CNS antigens in the draining lymph nodes, followed by infiltration of effector T cell subpopulations into the CNS through a now compromised BBB, their interaction with resident glial cells and culminating into heightened neuroinflammation, which finally opens gates for the infiltration of a second wave of immune cells into the CNS (Fletcher et al., 2010; Wilson et al., 2010). In addition to this, tissue repair mechanisms are also hampered due to the highly proinflammatory microenvironment. The neural tissue integrity is highly dependent on the ability of microglia and astrocytes to switch between homeostatic-resting, pro-inflammatory-damaging, and anti-inflammatory-repair phenotypes (Rothhammer and Quintana, 2015; Colonna and Butovsky, 2017; Liu et al., 2017). The phenotypic switch is regulated by the existing cytokine and growth factor milieu within the CNS. Due to their anatomical association microglia and astrocytes have different capacities to react to environmental cues.

Astrocytes are the most abundant cell type in the CNS. They are in close association with the BBB and neurons and hence are an important source of essential growth factors required for neurogenesis and neural homeostasis (Iadecola and Nedergaard, 2007). They can respond to danger signals and produce pro-and anti-inflammatory factors that can cause tissue damage or are involved in repair processes (Rothhammer and Quintana, 2015). Microglia are considered to be highly immune reactive cells of the CNS. They constantly sample the CNS microenvironment, sense the potential danger, and in the absence of an active immune system, provide factors that can potentially resolve the problem without causing much damage (Colonna and Butovsky, 2017). Their ability to phagocytose and clear the debris is essential for repair process (Sierra et al., 2013). However, their phenotype and function is altered under pathological conditions triggered by trauma, infection, or autoimmunity. Reactive astrogliosis and microgliosis, a common term used to refer to astrocyte and microglial activation, can have deleterious consequences. All these vital functions of microglia and astrocytes are regulated by a set of transcriptional regulators which, depending on the external stimuli, are activated and regulate gene expression.

CD4^+^ T helper cells mediate their effects by providing soluble factors that alter the behavior of other cells. Th1 and Th17 cells are among the key effector T helper cell subpopulations that have been closely associated with the pathology of MS. We have demonstrated in the past that soluble factors secreted by Th1 cells induced significant changes in the phenotype and function of both microglia and astrocytes (Prajeeth et al., 2014, 2017, 2018). However, Th17-derived effector molecules only acted on astrocytes and not on microglia. Although it was evident that Th1-derived effectors conferred a proinflammatory phenotype to microglia and astrocytes it was not clear how other functions were affected.

Global gene expression analysis of microglia and astrocytes exposed to Th1-derived culture supernatants revealed significant changes in the transcriptome of both cells types. Many genes were commonly regulated in microglia and astrocytes. However, a significant proportion of genes demonstrated cell type-specific regulation. In this study, we aimed to extract information of cell specific mechanisms induced by Th1-derived effectors that play a crucial role in the regulation of neuroinflammation and repair processes. We have previously characterized Th1-derived supernatants in terms of their cytokine profile and found IFN-γ and GM-CSF as the key cytokines present in high concentrations in these supernatants (Prajeeth et al., 2014). Therefore, it was not surprising that astrocytes and microglia exhibited the gene expression profile reminiscent of IFN-γ-mediated response. As listed in the ingenuity knowledge base, IFN-γ signaling regulates expression of *Nos2, Cxcl10, Icam1, Il6, Ptgs2, Cd40*, and *Ccl5*. Genes encoding these proteins were significantly upregulated in both microglia and astrocytes.

As a common feature Th1-derived effectors regulated the expression of genes associated with neuroinflammatory pathways both in microglia and astrocytes. This is well known and the findings here are just a confirmation of previously published works. However, a comparative study of the transcriptome induced by Th1-derived effectors in microglia and astrocytes is lacking, which would give us the information of how such effectors regulate other vital functions of these glial cell types relevant to neurodegeneration, repair, and regeneration. A recent study comparing responses of microglia and astrocytes to endotoxin treatment observed distinct transcriptional changes in these cells (Srinivasan et al., 2016). Similar to this study, a four-way plot analysis of DEGs revealed distinct transcriptional changes in microglia and astrocytes in response to Th1-derived effector molecules. In addition to analyzing a significant proportion of genes that were commonly regulated in these cell types we also focused our analysis on distinctly regulated genes. We used the IPA tool from Ingenuity systems and based our interpretations by comparing the canonical pathways, upstream regulators, diseases and functions, and regulator effects. The GPCR signaling pathway has been associated with the highest –log of *p*-value among the canonical pathways using the microglia-specific gene set. GPCRs play important roles in inflammation. Classic examples of receptors that signal through GPCR are the ones that bind to chemokines. Chemokines play a significant role in amplifying neuroinflammation (Mony et al., 2014; Le Thuc et al., 2015). They assist migration of microglia to the sites of inflammation. Furthermore, GPCR signaling also induces release of inflammatory mediators that play a role in the recruitment of other cells to the inflammatory foci. Apart from this, GPCR signaling has been implicated in the regulation of neuropathic pain. Interestingly, among the genes enriched in this pathway we also found transcripts that encode regulators of G-protein signaling (RGS). RGS are negative regulators of GPCR signaling. Expression of genes encoding RSG10 and RSG18 is downregulated in microglia by Th1-derived effectors. It has been shown that RGS10 promotes dopaminergic neuron survival via regulation of the microglial inflammatory response (Lee et al., 2008). Its knockdown in microglia resulted in dysregulated expression of inflammation-related genes (Lee et al., 2008). Another significant finding was that Th1-derived effectors downregulated several genes that regulate different stages of cholesterol biosynthesis. Cholesterol metabolism has a great importance in neurodegeneration associated with aging and MS. A recent study has demonstrated that clearance of cholesterol-rich myelin debris by phagocytosis is essential for regeneration (Cantuti-Castelvetri et al., 2018). Impaired clearance of myelin results in transition of free cholesterol into crystals and thereby inducing an immune response that impedes tissue regeneration (Cantuti-Castelvetri et al., 2018). Microglia are the professional phagocytes involved in myelin clearance in the CNS and this has been shown to have an impact on remyelination process (Skripuletz et al., 2013; Lampron et al., 2015). In the context of our findings, it is likely that Th1-derived effectors by inhibiting cholesterol metabolism might impede myelin uptake and regeneration. Phagocytosis is a key function of microglia which can be both beneficial and detrimental (Sierra et al., 2013). Another relevant finding in this regard is that the transcripts of tyrosine kinase phagocytic receptor MerTK were specifically upregulated in microglia. MerTK has been shown to be involved in myelin phagocytosis and its specific inhibitors reduced myelin phagocytosis (Healy et al., 2016). Hence it is likely that Th1-derived effectors influences the phagocytic function of microglia. In accordance with these findings we also identified phagosome formation pathways as top canonical pathway regulated by Th1-derived effectors in microglia.

Key canonical pathways that emerged from the analysis of DEG enriched specifically from astrocytes include positive regulation of the Eph/ephrin-mediated signaling pathway and inhibition of the FGF signaling pathway. Eph receptors are a tyrosine kinase family of receptors that binds to membrane bound ephrin ligands and mediate unique bidirectional signaling and known to assist astrocyte-neuron communication in the developing and adult CNS (Murai and Pasquale, 2011; Coulthard et al., 2012). Signals issued by Eph receptors and its ligands into their respective cells are referred to as forward and reverse signaling, respectively (Coulthard et al., 2012). Ephrin cues are presented in the form of concentration gradients and apparently play a role in guiding axonal growth. We found genes encoding EphA5 and EphB3 upregulated and EphB1 and EphB6 downregulated in astrocytes. Therefore, it is likely that Th1-derived effectors modulate neural regeneration through astrocytes. However, from the existing evidence it is difficult to conclude if astrocytes in response to Th1-derived effectors assist or suppress neurogenesis. In addition, our data also predict that Th1-derived effectors are likely to impact neural tissue regeneration by downregulating expression of several members of the FGF family in astrocytes. FGF are growth factors which play an important role in neuronal and glial cell differentiation (Turner et al., 2016). FGF signaling in the CNS has both beneficial and detrimental effects. A recent study has shown that FGF9 inhibits remyelination and induces a proinflammatory environment in MS lesions (Lindner et al., 2015). We observed that expression of genes encoding FGF9 was suppressed in astrocytes in response to Th1-derived effectors. Other studies have shown that FGF inhibits reactive astrogliosis and might improve axonal regeneration and functional recovery (Kang et al., 2014).

The information about the possible biological impact from this transcriptome analysis came from studying the regulator effects in IPA. It appears that Th1-derived effector molecules mainly regulate the pathways that impact cell-cell communication, cell migration activation, and repair processes in astrocytes and microglia. One of the key effectors that regulate the above mentioned processes are the cytokines and growth factors. Our comparative analysis of genes encoding cytokines and growth factors enriched from 3170 DEGs between astrocytes and microglia suggests that in response to Th1-derived effectors predominantly microglia provide cytokines that regulate activation and migration of cells into the inflammatory sites. Astrocytes also regulate migration of cells in to the CNS by upregulating chemokines and cell adhesion molecules. However, the most distinct effect was the more readily downregulated expression of several crucial growth factors in astrocytes as compared to microglia. This is in support of growing evidence that a dysregulated function of astrocytes might hamper repair process following neuroinflammatory damage (Toft-Hansen et al., 2011; Skripuletz et al., 2013). Although we identify several genes governing various cellular processes to be specifically regulated in a particular glial cell type in response to Th1-derived factors, one cannot rule out the possibility that these genes may follow different expression kinetics in microglia and astrocytes. We have tested this for few genes encoding chemokines and growth factors and found that at least Cx3cl1 and Gdnf expression was regulated at different time points in microglia and astrocytes. Hence it would be vital for further studies aiming to understand the effects of T cells effectors on particular microglial or astrocyte function to also include time kinetics.

In conclusion, our comparative transcriptome analysis in microglia and astrocytes shed light on distinct pathways regulated by Th1-derived effectors in these glial cell types. We strongly believe our findings are highly relevant for studies aiming at understanding T cell mediated neuropathologies with focus beyond cytokines and growth factors and to explore other aspects such as metabolic pathways regulated by effector T cells in glial cells that may potentially influence neurodegeneration and repair processes. This may eventually lead to new therapeutic targets and devising new strategies to treat neuroinflammatory and neurodegenerative disorders.

## Author contributions

CP, JH, and MS conceived the experiments. CP performed experiments. OD-B performed microarrays. CP, OD-B, and ST analyzed microarray data. JH helped with Th1- and Th17 cultures and generated supernatants. PR helped with the cytokine profiling of Th1 culture supernatants. CP and MS wrote the manuscript.

### Conflict of interest statement

The authors declare that the research was conducted in the absence of any commercial or financial relationships that could be construed as a potential conflict of interest.

## Supplementary material

Supplementary Figure 1 Purity of cells generated with our culture protocols. Representative images show the purity of microglia and astrocyte harvested from neonatal mouse brain mixed glial cell preparations. **(A)** Microglia harvested by short agitation (30 min at 180 rpm) of cultrure flasks yielded highly pure microglia (CD11b^+^ cells) as assessed by flow cytometry. **(B,C)** Astrocyte cultures obtained after eliminating reminiscent microglia and oligodendrocyte precursor cells by overnight agitation (170 rpm) followed by AraC treatment had high percentage of GFAP^+^ astrocytes and very few contaminating microglia (CD11b^+^ and Iba1^+^ cells). **(D)** Polarized Th1 cells were either unstimulated of restimulated with anti-CD3/CD28 and its purity was assessed by staining for IFNγ and IL-17A. Dot plots show that polarized Th1 cells contained high percentage of IFNγ^+^ IL-17^−^ cells. **(E)** Concentration of mouse Th cytokines was measured in the Th1 culture supernatants. Data presented here is mean ± SD of culture supernatants collected from four independent Th1 cultures (*n* = 4).

Supplementary Table 1 Genes regulated by IFN-g/STAT1 signaling.

Supplementary Table 2 Taqman probes used for RT-PCR.
