# Supplementary figures and images for: IFN-γ Producing Th1 Cells Induce Different Transcriptional Profiles in Microglia and Astrocytes
Source: Front Cell Neurosci. 2018 Oct 10;12:352. doi: 10.3389/fncel.2018.00352 (PMC6191492; doi:10.3389/fncel.2018.00352)

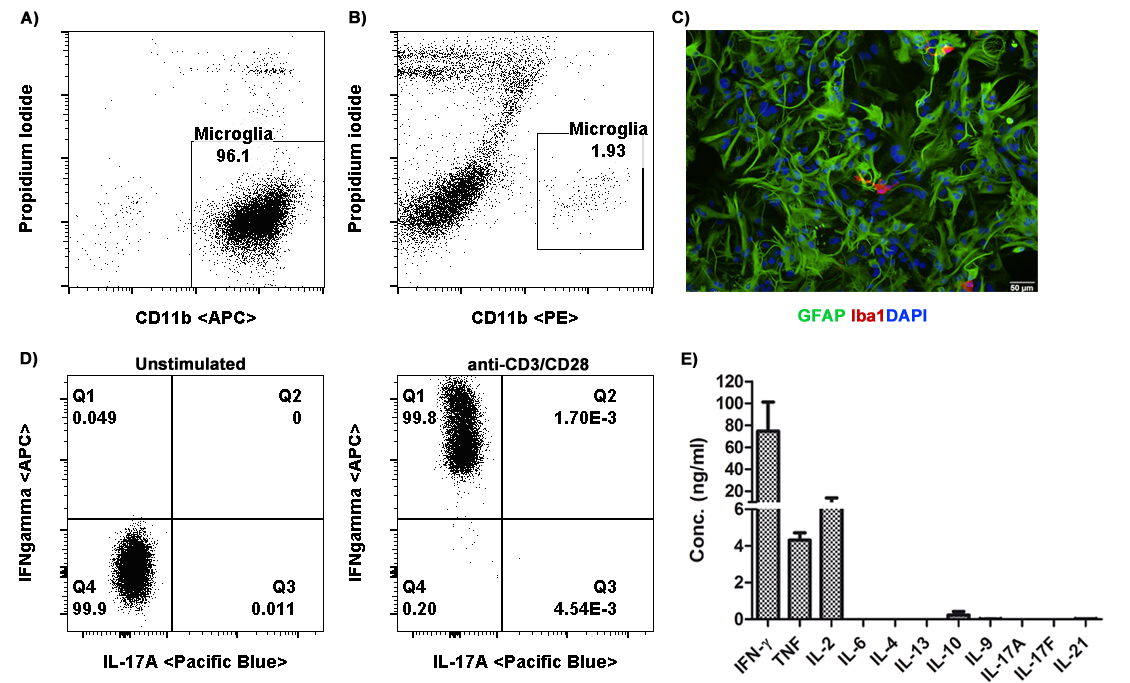

Supplement: Supplementary Figure 1 — Purity of cells generated with our culture protocols. Representative images show the purity of microglia and astrocyte harvested from neonatal mouse brain mixed glial cell preparations. (A) Microglia harvested by short agitation (30 min at 180 rpm) of cultrure flasks yielded highly pure microglia (CD11b+ cells) as assessed by flow cytometry. (B,C) Astrocyte cultures obtained after eliminating reminiscent microglia and oligodendrocyte precursor cells by overnight agitation (170 rpm) followed by AraC treatment had high percentage of GFAP+ astrocytes and very few contaminating microglia (CD11b+ and Iba1+ cells). (D) Polarized Th1 cells were either unstimulated of restimulated with anti-CD3/CD28 and its purity was assessed by staining for IFNγ and IL-17A. Dot plots show that polarized Th1 cells contained high percentage of IFNγ+ IL-17− cells. (E) Concentration of mouse Th cytokines was measured in the Th1 culture supernatants. Data presented here is mean ± SD of culture supernatants collected from four independent Th1 cultures (n = 4). [file Image_1.TIF]
